# Cigarette smoke but not electronic cigarette aerosol activates a stress response in human coronary artery endothelial cells in culture

**DOI:** 10.1016/j.drugalcdep.2016.04.020

**Published:** 2016-06-01

**Authors:** Jack E. Teasdale, Andrew C. Newby, Nicholas J. Timpson, Marcus R. Munafò, Stephen J. White

**Affiliations:** a School of Clinical Sciences, University of Bristol, Bristol, UK; b MRC Integrative Epidemiology Unit at the University of Bristol, Bristol, UK; c School of Social and Community Medicine, University of Bristol, Bristol, UK; d UK Centre for Tobacco and Alcohol Studies, School of Experimental Psychology, University of Bristol, Bristol, UK

**Keywords:** Cigarettes, E-cigarettes, Human coronary artery cells, Stress response

## Abstract

**Background:**

It is generally acknowledged that e-cigarettes are unlikely to be as harmful as conventional cigarettes, but there is little data that quantifies their relative harms. We investigated the biological response to e-cigarette aerosol exposure (versus conventional cigarette smoke exposure) at the cellular level, by exposing human coronary artery endothelial cells (HCAEC) to aqueous filtered extracts of e-cigarette aerosol or cigarette smoke and looking at gene expression changes consistent with a stress response. This included genes controlled by the oxidant-stress sensing transcription factor NFR2 (NFE2L2), and cytochrome P450 family members.

**Methods:**

Cigarette smoke extract (CSE) was created using mainstream smoke from a single cigarette drawn through 10 ml of endothelial cell growth media MV2. Electronic cigarette aerosol extract (eCAE) was created using the same apparatus, using a constant power output of 10.8 w (4.2 V) and 18 mg/ml nicotine solution. eCAE was generated using 5 cycles of 5 s heat with at least 10 s in between each puff to allow the coil to cool, air being drawn through the device at 70 ml/minute.

**Results:**

HCAEC responded to the noxious components in CSE, resulting in activation of NRF2 and upregulation of cytochrome p450. However, eCAE did not induce NRF2 nuclear localisation, upregulation of NRF2-activated genes, or the upregulation of cytochrome p450.

**Conclusions:**

The use of e-cigarettes as a substitute for conventional cigarettes is likely to reduce immediate tobacco-related harm, at least with respect to cardiovascular harms.

## Highlights

• Human coronary artery endothelial cells show a biological response to cigarette smoke.
• This response was not seen following exposure to e-cigarette aerosol.
• Using e-cigarettes instead of cigarettes may reduce immediate cardiovascular harms.

## 1 Introduction

The rapid growth in the use of e-cigarettes, which deliver nicotine via inhaled aerosol rather than tobacco smoke, has generated debate regarding their potential benefits relative to conventional cigarettes. While e-cigarettes are unlikely to be as harmful as conventional cigarettes, there is little data that quantifies their likely relative harms, and data does not yet exist on the correlates of long-term e-cigarette use. Given this, the development of laboratory models that quantify the biological effects, and therefore likely relative harms, of e-cigarettes and conventional cigarettes is critical.

The levels of chemicals known to be harmful present in e-cigarette aerosol depend on several variables such as the solution used and the battery output voltage (Bahl et al., 2012, Kosmider et al., 2014). Critically, a number of these experiments have been performed with diluted refill solutions (e-liquid), rather than heated aerosol (Bahl et al., 2012). The levels of toxic product will depend on the way the e-cigarette is used (Farsalinos et al., 2015). Moreover, the mere presence of detectable levels of potentially harmful chemicals does not necessarily indicate that e-cigarette aerosol will deliver concentrations required for the chemical to be toxic. In the absence of long-term prospective data on the health correlates of e-cigarette use, there is a need to investigate the potential harmful effects of e-cigarette use. One valuable approach might be to examine the biological response of human primary cells to e-cigarette aerosol.

The oxidant-stress sensing transcription factor NFR2 (nuclear factor, erythroid 2-like 2, NFE2L2) is normally sequestered in the cytoplasm through interaction with kelch-like ECH-associated protein 1 (KEAP1), but this interaction is disrupted upon electrophilic attack, allowing NFR2 to translocate to the nucleus and activate gene expression (Müller and Hengstermann, 2012). As such, activation of the NRF2 system is a useful cellular biomarker of biologically relevant levels of free radicals. Combustion products of tobacco activate NFR2-regulated gene expression in the lung and initiate a transcription profile that contributes to protection from the stress induced by tobacco smoke (Rangasamy et al., 2004, Iizuka et al., 2005, Müller and Hengstermann, 2012). The role of NRF2 in the vasculature is less clear; modest activation by physiological laminar flow is thought to protect endothelial cells from oxidative stress (Zakkar et al., 2009). However, when crossed with a hyperlipidaemic mouse, the Nrf2 knockout mouse develops less atherosclerosis, suggesting NRF2 activation contributes to disease progression (Barajas et al., 2011). The upregulation of cytochrome P450 family members by cigarette smoke has also been reported previously, indicating they are another biomarker of toxic compounds found in cigarette smoke (Baker et al., 2001, Zevin and Benowitz, 1999).

We investigated the biological response to e-cigarette aerosol exposure and conventional cigarette smoke exposure at the cellular level, by exposing primary human coronary artery endothelial cells to aqueous filtered extracts of aerosol or smoke and measuring gene expression changes consistent with a stress response.

## 2 Methods

### 2.1 Generation of cigarette smoke extract and electronic cigarette aerosol extract

Cigarette smoke extract (CSE) was created using mainstream smoke from a single Marlboro Gold cigarette (7 mg tar, 0.6 mg nicotine) drawn through 10 ml of endothelial cell growth media MV2 (Promocell, C-22120) at a rate of 70 ml/min. Under these conditions, the cigarette was consumed in ∼5.5 min resulting in ∼385 ml of mainstream smoke being drawn through the solution. Electronic cigarette aerosol extract (eCAE) was created using the same apparatus, using an iStick battery at constant power output (10.8 W, 4.2 V), with an Aerotank Mini atomiser (1.8 Ω) loaded with Haven fluid USA Mix 18 mg/ml nicotine solution (80% vegetable glycerine, 20% propylene glycol). eCAE was generated using 5 cycles of 5 s heat with at least 10 s in between each puff to allow the coil to cool, with air being drawn through the device at 70 ml/min. A visible vapour was generated using these conditions. A fresh pre-soaked heating coil was used for each experiment. A higher power output was selected for generation of eCAE as it has been shown that the levels of potentially harmful chemicals produced by e-cigarettes increases with voltage applied to the heating coil (Kosmider et al., 2014).

CSE and eCAE were filtered using a 0.2 μM sterile filter to remove any particulate matter (to sterilise the extracts, and also to simulate the filtering that would occur in the lung), and diluted to 10% in endothelial cell growth media before being added to the cell. CSE and eCAE were used immediately after generation. Nicotine concentrations in CSE and eCAE were measured by ABS Laboratories Ltd, which indicated that both contained 3.5 μg/ml of nicotine; therefore, the diluted solutions applied to the cells contained 350 ng/ml nicotine. Nicotine (Sigma, N3876) was diluted to 350 ng/ml and used as a control.

### 2.2 Tissue culture

Human coronary artery endothelial cells (HCAEC) from 3 to 6 different donors, passage 4–5, were seeded in 6 well plates and grown to form a confluent monolayer. They then received 3 sequential treatments of 10% CSE, 10% eCAE, or nicotine (350 ng/ml) 16 h apart, to study the stable effects of these treatments on cells (see supplementary methods and model validation data). Cells were lysed 16 h after the last treatment and total RNA isolated using Purelink RNA mini kit (Ambion, Life technologies) for analysis of gene expression changes.

**Fig. 1:**
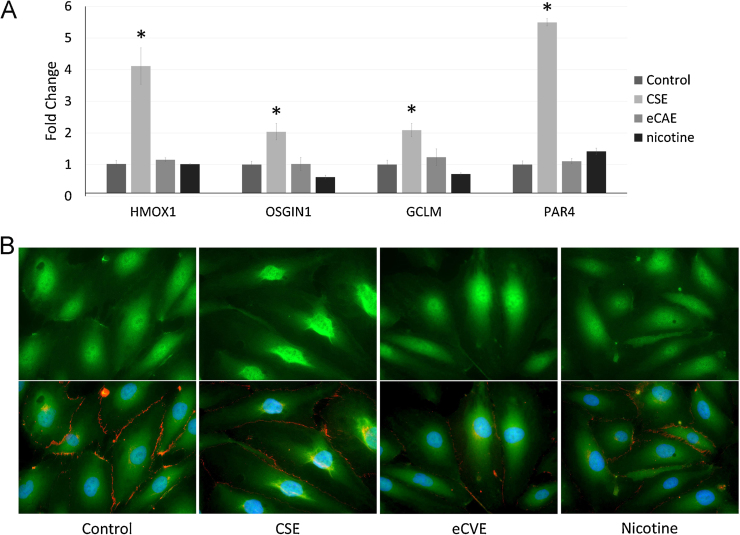
CSE but not eCAE or nicotine increase NRF2-regulated gene expression. Panel A) Fold change of mRNA expression levels of heme oxygenase 1 (*HMOX1*), oxidative stress growth inhibitor 1 (*OSGIN1*), glutamate-cysteine ligase (*GCLM*) and protease activated receptor 4 (PAR4–from *F2RL3* gene), exposed to 3 sequential treatments of CSE (10%), eCAE (10%) or nicotine (350 ng/ml, 2.16 μM), or vehicle control (*P < 0.05 v all other treatments, n = 4–6). Panel B) Cellular localisation of NRF2 as assessed by immunocytochemistry 2 h after a single treatment. CSE induced a shift in localisation to a predominantly nuclear localisation, co-localising with the nuclear blue dapi staining (bottom row of panel B). Error bars represent the SEM. Numerical results are shown in Supplementary Table 2.

We independently validated a panel of NRF2-regulated genes in HCAEC using adenoviral overexpression of NRF2 (see Supplementary Fig. 1) and used this panel of genes to monitor NRF2-regulated gene expression in response to treatment (Fig. 1A).

### 2.3 Analysis of gene expression

Quantitative PCR (qPCR) was performed on 500 ng reverse transcribed total RNA using QuantiTect Reverse Transcription Kit (Qiagen) with LightCycler 480 SYBR Green I Master Mix (Roche) using primers listed in Supplementary Table 1. All replicates were performed using primary cells from different donors. Statistical analyses were performed using a oneway ANOVA with Tukey-Kramer post hoc tests.

### 2.4 Immunocytochemistry

Immunocytochemistry to detect NRF2 localisation was performed on HCAEC exposed to treatments for 2 h, before being fixed with 4% paraformaldehyde for 10 min. NRF2 localisation was detected using rabbit anti-Nrf2 (Santa Cruz SC-722) and cell boundaries using mouse anti-βcatenin (BD 610153).

## 3 Results

We selected genes for investigation (*SRXN1*, *DDIT4L*, *HMOX1*, *GCLM*, *OSGIN1*, *PAR4*, *CYP1A1, CYP1B1*) on the basis of literature searches and previous unpublished work. From these, we selected six (*HMOX1*, *GCLM*, *OSGIN1*, *PAR4*, *CYP1A1*, *CYP1B1)* that showed clear evidence of regulation by CSE, defined as greater that two-fold relative to control, and compared these to both nicotine and eCAE.

### 3.1 CSE but not eCAE activates the oxidative stress pathway

Upregulation of *HMOX1*, *GCLM*, *OSGIN1* and *PAR4* was observed as following exposure to CSE (P < 0.05 v all other treatments, n = 3–6), but not by eCAE or nicotine. This suggests NRF2 is activated by CSE, but not eCAE. Immunocytochemistry was used to analyse the intracellular location of NRF2. Two-hour exposure to CSE induced a shift in NRF2 from a predominantly cytoplasmic to a predominantly nuclear localisation, indicating NRF2 activation (Fig. 1B).

### 3.2 CSE but not eCAE increases expression of cytochrome P450, IL8 and NTPX1

**Fig. 2:**
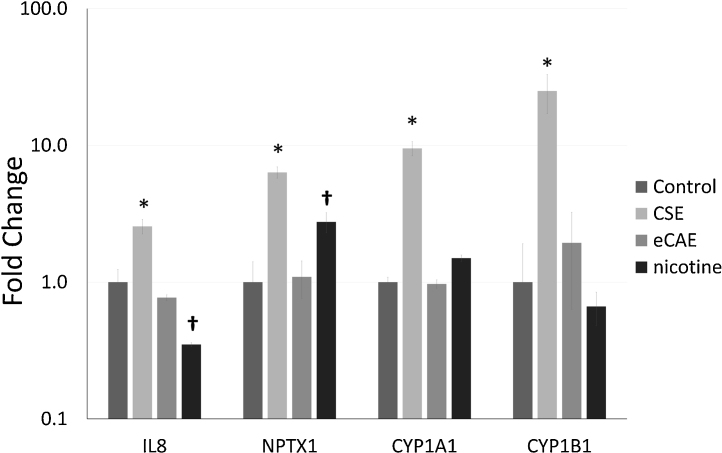
Changes in gene expression in interleukin 8, neuronal pentraxin I and cytochrome p450 1A1 and 1B1. Changes in gene expression in interleukin 8 (*IL8*), neuronal pentraxin I (*NPTX1*) and cytochrome p450 1A1 and 1B1 (*CYP1A1, CYP1B1*) exposed to 3 sequential treatments of CSE (10%), eCAE (10%) or nicotine (350 ng/ml, 2.16 μM), or vehicle control (*P < 0.05 vs all other treatments, n = 3–6; †P < 0.05 vs eCAE, n = 3–6). Change is shown on a logarithmic scale (base 10). Error bars represent the SEM. Numerical results are shown in Supplementary Table 2.

Of the different p450 cytochromes expressed in HCAEC, two (*CYP1A1*, *CYP1B1*) were upregulated by CSE (Fig. 2, P < 0.05 vs all other treatments, n = 3–6); however, eCAE did not affect gene expression levels. We also assessed interleukin 8 (*IL8*) and neuronal pentraxin I (*NTPX1*), because we have found both to be regulated by CSE (unpublished data). We observed that CSE upregulated the expression of both *IL8* and *NTPX1*, while eCAE did not. Interestingly, *IL8* expression was reduced and *NTPX1* increased by nicotine, which were unchanged by eCAE despite equivalent nicotine concentrations (P < 0.05 vs eCAE, n = 3–6).

## 4 Discussion

We explored gene expression changes consistent with a cellular response to perceived xenobiotic stress, activation of the antioxidant response and increases in cytochrome p450, in response to CSE and eCAE. Our results indicate that HCAEC respond to the noxious components in CSE, resulting in activation of NRF2 and upregulation of cytochrome p450 (i.e., a stress response). However, eCAE did not induce NRF2 nuclear localisation, upregulation of NRF2-activated genes, or the upregulation of cytochrome p450. *IL8* directly enhances endothelial cell survival, proliferation, and matrix metalloproteinases production (Li et al., 2003). *NPTX1* is a novel epigenetic regulation gene associated with lung cancer prognosis (Zhou et al., 2015) that enhances the level of endothelial apoptosis (Guzeloglu-Kayisli et al., 2014). Interestingly, there was a difference in expression of both of these genes between nicotine and eCAE, despite an equal concentration of nicotine in both, suggesting that while eCAE does not induce the same stress response as CSE, there is a biological effect of other constituents of eCAE, albeit one that does not reflect a stress response in our model.

This study used primary HCAEC because of their involvement in the development of cardiovascular disease (Sima et al., 2009, Gimbrone Jr. and García-Cardeña, 2013); additionally, we attempted to create conditions relevant to this investigation by bubbling filtered mainstream smoke through tissue culture media, which was filtered (0.2 μm) and diluted before being added to cells. Whilst it is not possible to perfectly simulate the mode and systemic effects of smoking, CSE contained only the soluble components of cigarette smoke gas phase and not the tar phase or particulates >0.2 μm. This made conditions more suitable for looking at the systemic vascular effects of cigarette smoke, as opposed to the lungs. The use of tissue culture media may be important as it contains 5% serum. Highly reactive chemical moieties and free radicals may react with and modify the proteins in the media to create proinflammatory adducts, which in turn may transmit some of the noxious effect of CSE to the cell, similar to the way these reactants may modify plasma proteins in vivo. The volume of smoke used in our model corresponds to roughly half of the smoke inhaled from a low tar cigarette (7–8 puffs) by a regular smoker (Djordjevic et al., 2000, Zacny and Stitzer, 1996). eCAE was generated in an identical manner to CSE, resulting in the same concentration of nicotine in the extract (3.5 μg/ml).

There are some limitations to this study that should be considered. First, we investigated a single e-cigarette product, a single cell line, and a limited number of outcomes. Therefore, our results should be considered preliminary, and their generalizability tested in future studies across a range of products, fluids, device settings, cell lines and outcomes. Second, our puffing protocols were not designed to directly mimic real-world smoking or vaping, but to standardise nicotine exposure. Future work should apply protocols that mimic real-world behaviour. Third, the constant flow rate used to produce CSE means that spikes in combustion temperature will have been minimal. These spikes, generated by puffing, generate large yields of combustion products, meaning the effects of CSE we observed may in fact be conservative. Fourth, we only examined the effects of CSE and eCAE separately. Given that dual-use of conventional cigarettes and e-cigarettes is common, future models should test different combinations of exposure.

Our results suggest that the use of e-cigarettes as a substitute for conventional cigarettes is likely to reduce immediate tobacco-related cardiovascular harms. The absence of NRF2 activation or upregulation of cytochrome p450 in HCAEC suggests a lower biological impact of eCAE compared to CSE. This is in agreement with other studies suggesting a lower potential disease burden for e-cigarette use compared with conventional cigarettes (Oh and Kacker, 2014, Goniewicz et al., 2014). In the absence of long-term prospective data on the health correlates of e-cigarette use, there is a critical need to valid laboratory models on the relative harms of e-cigarettes and conventional cigarettes to inform the ongoing public health debate regarding their use. This study identifies two biological axes for analysing the components of CSE and eCAE, and indicates CSE but not eCAE induces a stress response in human cells.

## Conflict of interest

None.

## Funding

Nothing declared.

## Contributors

Jack E. Teasdale performed the experiments, and approved the final manuscript for submission.

Andrew C. Newby conceived the study, and approved the final manuscript for submission.

Nicholas J. Timpson conceived the study, and approved the final manuscript for submission.

Marcus R. Munafò conceived the study, drafted the manuscript, and approved the final manuscript for submission.

Stephen J. White conceived the study, supervised the experiments, drafted the manuscript, and approved the final manuscript for submission.
