# Supplementary material for: Cigarette smoke but not electronic cigarette aerosol activates a stress response in human coronary artery endothelial cells in culture
Source: Drug Alcohol Depend. 2016 Jun 1;163:256–60. doi: 10.1016/j.drugalcdep.2016.04.020 (PMC4907307; doi:10.1016/j.drugalcdep.2016.04.020)
Supplement: Supplementary file 1 [file mmc1.docx]

**Supplementary Material for the article**

Cigarette smoke but not electronic cigarette aerosol activates a stress response in human coronary artery endothelial cells in culture

Jack E Teasdale ^1^, Andrew C Newby ^1^, Nicholas J Timpson ^2,3^, Marcus R Munafò ^2,4^ *, Stephen J White ^1 *^

1. School of Clinical Sciences, University of Bristol, Bristol, UK.

2. MRC Integrative Epidemiology Unit at the University of Bristol, Bristol, UK.

3. School of Social and Community Medicine, University of Bristol, Bristol, UK.

4. UK Centre for Tobacco and Alcohol Studies, School of Experimental Psychology, University of Bristol, Bristol, UK.

* Joint senior author

Corresponding author: Marcus R. Munafò, School of Experimental Psychology, University of Bristol, 12a Priory Road, Bristol BS8 1TU, United Kingdom. T: +44.117.9546841; F: +44.117.9288588; E: [marcus.munafo@bristol.ac.uk](mailto:marcus.munafo@bristol.ac.uk)

**This material supplements, but does not replace, the peer-reviewed article in**

**Drug and Alcohol Dependence.**

**Supplementary Methods**


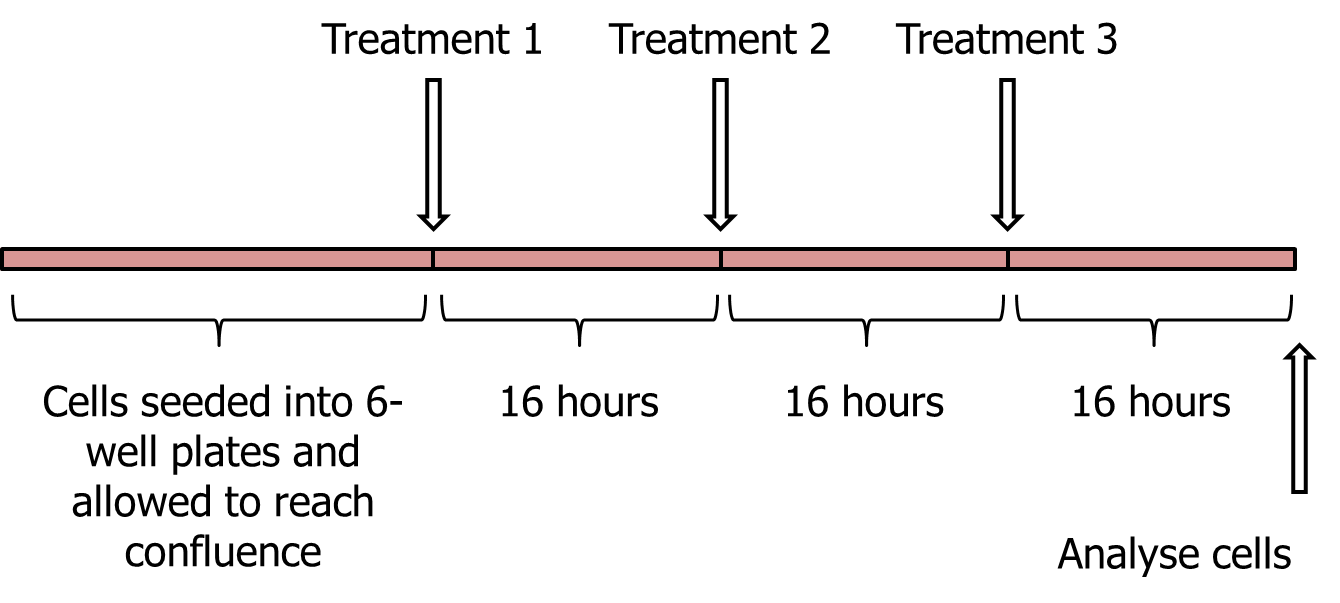


Human coronary artery endothelial cells (HCAEC) purchased from PromoCell and grown in Endothelial cell growth media MV2 (C-22121, PromoCell). Cells from at least 3 different donors were used for each experiment, at passage 4 or 5. HCAEC were seeded in 6 well plates and allowed to grow to form a confluent monolayer. They then received 3 sequential treatments of 10% CSE, 10% eCAE, or nicotine (350 ng/ml) 16 hours apart, to study the stable effects of these treatments on cells. The control for CSE or eCVE was unmodified media, while the control for nicotine was a matched dilution of ethanol (1/1000), as it was initially solubilised in 100% ethanol. Cells were washed with PBS and lysed 16 hours after the last treatment. HCAEC from a separate batch of cells were used for each experimental replicate. Each batch of HCAEC came from a different donor, individual batches were kept separate and not pooled, to assess the inter-donor differences in response.

The CSE was generated from roughly half of the smoke inhaled from a low tar cigarette (7 to 8 puffs) by a regular smoker in 10 ml of media. This was then diluted 10-fold, which would be equivalent to 100 ml (therefore a whole cigarette would generate 200 ml of CSE). The average blood volume is approximately 5 litres, so using the CSE at 10% is roughly equivalent to 25 cigarettes, which we applied every 16 hours.

We made an extract from the aqueous, filtered phase of cigarette smoke and employed this in a model we see as important in the investigation of a systemic response to smoke. The more harmful components of electronic cigarette vapour are created at higher power settings. We therefore selected a high power setting to create a ‘worst-case’ extract. We titrated the dose until we matched the nicotine level in the cigarette smoke extract on the assumption that a user would inhale a dose of vapour until they had received the same nicotine levels that they would have received from an equivalent amount of cigarette smoke.

**Supplementary Figure 1. Validation of NRF2 regulation using overexpression of NRF2.**


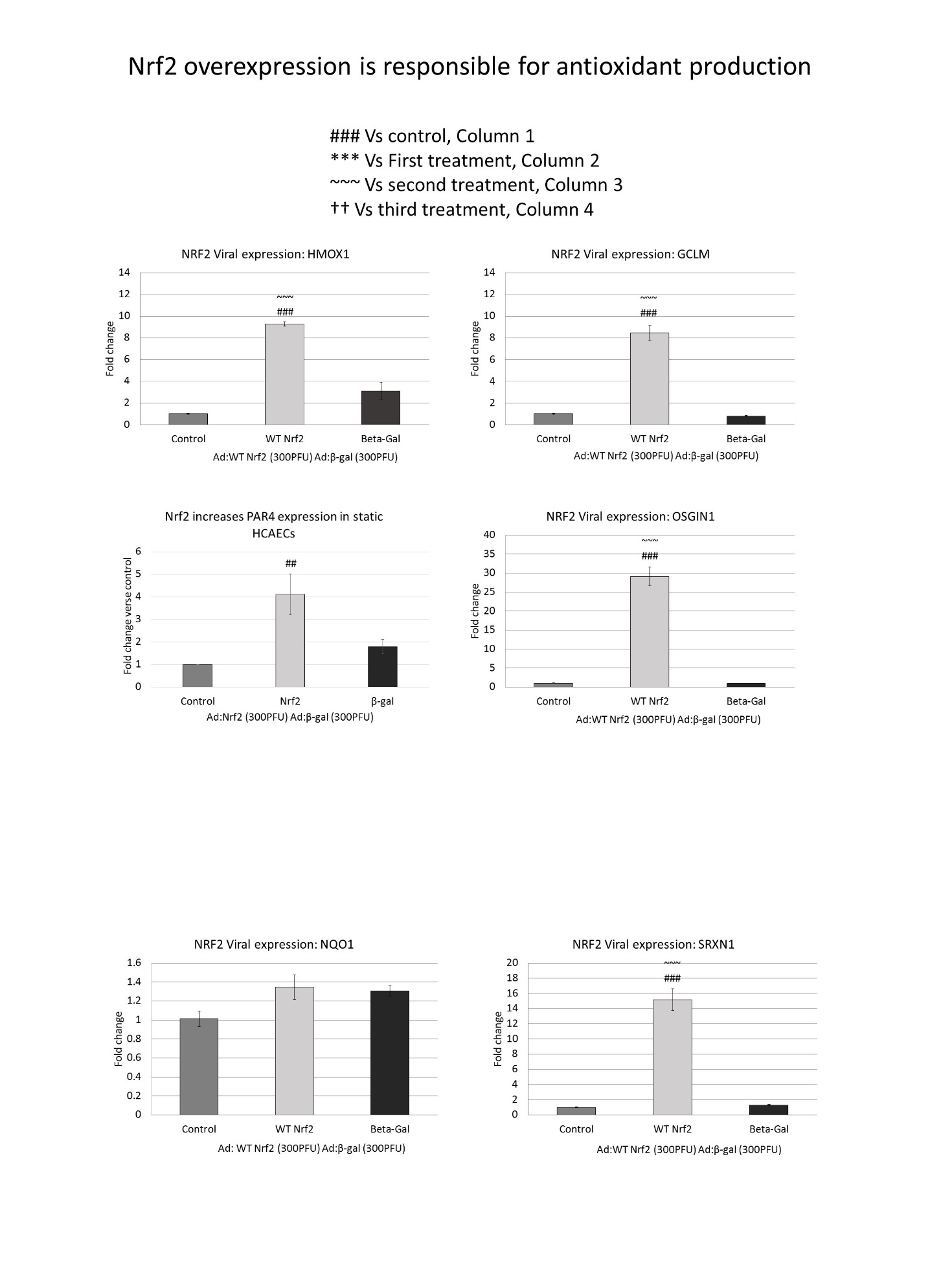


Human coronary artery endothelial cells were transduced with adenoviral vector expressing either wild type Nrf2 (WT Nrf2) or β-galactosidase (Beta-Gal) at 300pfu/cell. 48 hours post transduction, cells were lysed, RNA purified and gene expression analysed by qPCR (# P < 0.05, ## P < 0.01, ### P < 0.001 v control; ~ P < 0.05, ~~ P < 0.01, ~~~ P < 0.001 v β-galactosidase control). Error bars represent SE.

**Supplementary Table 1. List of Primers.**

| Gene name | Primer number | Primer sequence |
| --- | --- | --- |
| *HMOX1* | SW226F | TCAGGCAGAGGGTGATAGAAGAGG |
|  | SW227R | GCCACCAGAAAGCTGAGTGTAAGG |
| *OSGIN1* | SW746F | GGGAGCCTGGCACTCCATCG |
|  | SW747R | CCCGGCTGTTGCGAAGACCT |
| *GCLM* | SW754F | GTCCTTGGAGTTGCACAGCTGGA |
|  | SW755R | GGCATCACACAGCAGGAGGCA |
| *PAR4* | V5 | GGCAACCTCTATGGTGCC |
|  | V5 | TTCGACCCAGTACAGCCTTC |
| *IL8* | SW313F | TGGCTCTCTTGGCAGCCTTC |
|  | SW314R | CCCAGTTTTCCTTGGGGTCC |
| *NTPX1* | SW813F | GGGCACGCCCTTCTCCTACG |
|  | SW814R | TAGGCCTCCCAGACCCCGTC |
| *CYP1A1* | SW817F | AGCAGCTGGATGAGAACGCCA |
|  | SW818R | CGCCGTGACCTGCCAATCACT |
| *CYP1B1* | SW823F | TGACATCTTCGGCGCCAGCC |
|  | SW824R | ACAAGGCAGACGGTCCCTCC |

Quantitative PCR (qPCR) was performed on 500 ng reverse transcribed total RNA using QuantiTect Reverse Transcription Kit (Qiagen) with LightCycler 480 SYBR Green I Master Mix (Roche) with a 62 degree anneal temperature. qPCR products all demonstrated a single peak in the melt curve and one product of the expected size when resolved on an agarose gel.

**Supplementary Table 2. Fold-change in gene expression in human coronary artery endothelial cells**

|  |  | ***HMOX1*** | ***OSGIN1*** | ***GCLM*** | ***PAR4*** | ***IL8*** | ***NPTX1*** | ***CYP1A1*** | ***CYP1B1*** |
| --- | --- | --- | --- | --- | --- | --- | --- | --- | --- |
| **Control**  **(n = 4)** | Change | 1.00 | 1.00 | 1.00 | 1.00 | 1.00 | 1.00 | 1.00 | 1.00 |
|  | 95% CI | (0.61 to 1.39) | (0.67 to 1.33) | (0.56 to 1.44) | (0.58 to 1.42) | (0.24 to 1.76) | (-0.31 to 2.31) | (0.72 to 1.28) | (-1.91 to 3.91) |
| **Nicotine**  **(n = 3)** | Change | 1.01 | 0.60 | 0.70 | 1.42 | 0.35 | 3.04 | 1.50 | 0.66 |
|  | 95% CI | (0.91 to 1.11) | (0.36 to 0.84) | (0.53 to 0.87) | (0.90 to 1.94) | (0.29 to 0.41) | (1.02 to 5.05) | (1.16 to 1.84) | (-0.12 to 1.44) |
| **eCAE**  **(n = 5)** | Change | 1.13 | 1.02 | 1.23 | 1.11 | 0.77 | 1.10 | 0.97 | 1.94 |
|  | 95% CI | (0.95 to 1.31) | (0.44 to 1.59) | (0.51 to 1.96) | (0.84 to 1.37) | (0.66 to 0.89) | (0.06 to 2.13) | (0.81 to 1.14) | (-1.69 to 5.57) |
| **CSE**  **(n = 6)** | Change | 4.03 | 2.04 | 2.09 | 5.50 | 2.56 | 6.36 | 9.52 | 25.05 |
|  | 95% CI | (2.45 to 5.61) | (1.37 to 2.71) | (1.54 to 2.63) | (3.84 to 7.16) | (1.69 to 3.44) | (4.66 to 8.06) | (7.28 to 11.75) | (4.59 to 45.52) |

Control is the reference. 95% CI: 95% confidence interval; eCAE: electronic cigarette aerosol extract; CSE: cigarette smoke extract.

**Model validation data**

In order to establish the appropriate dose of CSE to apply to cells, a number of different assays were performed to assess the effects of different doses of CSE. In the majority of experiments, TNFα was also included as a positive control for the assays involved. Figures in this section are prefixed V- to indicate they are part of the validation data.

**TNFα and CSE treatment on static HCAECs to discern the dose of future experimental conditions**

***TNFα, but not CSE, increases VCAM1 expression in static HCAECs***

Increased VCAM1 expression and ROS production are an established consequence of endothelial dysfunction in the vasculature, and data indicates this process is potentiated by TNFα [1-4]. For the initial experiments we investigated the effects of TNFα and CSE on static HCAECs to attain the appropriate dose and time points of future work. Under these conditions, TNFα stimulated a significant dose-dependent increase in VCAM1 (Figure V-1A), whereas CSE did not affect VCAM1 at any concentrations (Figure V-1B). Interestingly, when added together (Figure V-2) TNFα and CSE produced a significantly increased VCAM1 response, compared to that of TNFα and a treatment of TNFα and H_2_O_2_ (as an source of ROS). This implies that under static conditions TNFα and CSE synergise to increase VCAM1 production.


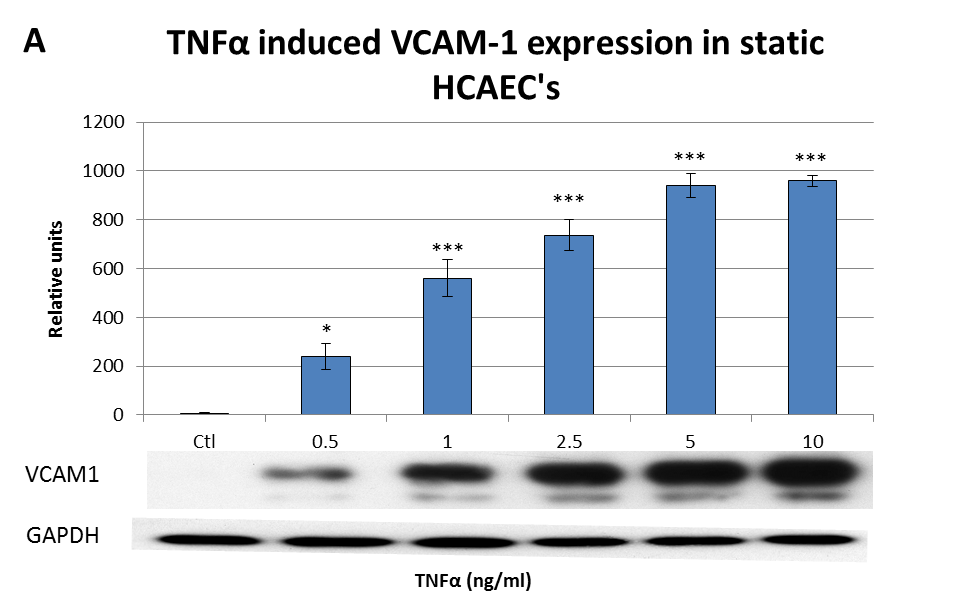

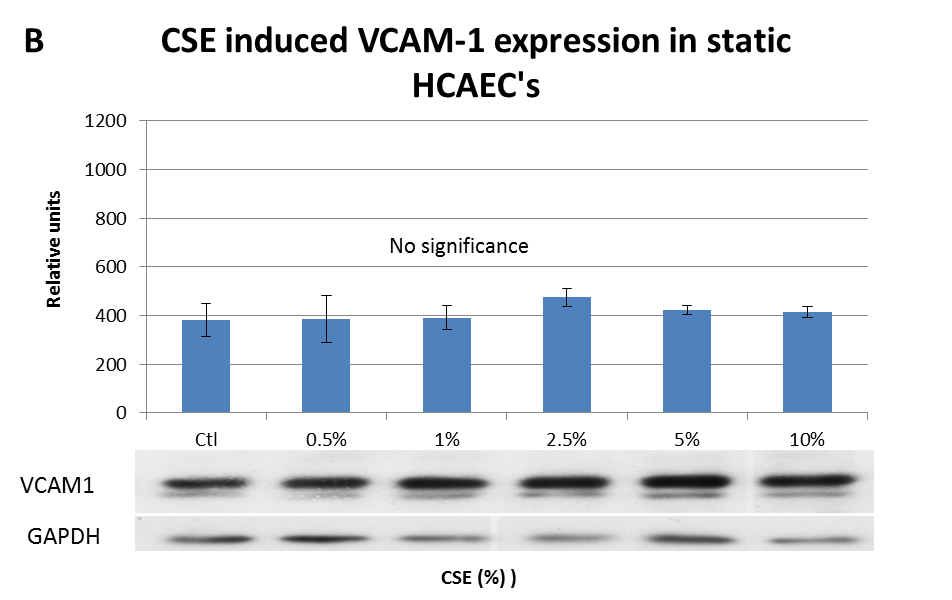


**VCAM1 protein expression in static HCAECs exposed to varying concentrations of CSE for 24 hours**

**VCAM1 protein expression in static HCAECs exposed to varying concentrations of TNFα for 24 hours**

**Relative optical density**

**Relative optical density**

Figure V-1 TNFα induces endothelial dysfunction in static HCAECs. A: 24 hr exposure to TNFα increases VCAM1 production in a dose dependent manner in static HCAECs, in media minus supplements. * P < 0.05, *** P <0.001 v control. B: 24 hr exposure to CSE does not change VCAM1 production in static HCAECs at any concentration. 15,000 cells loaded. Statistical tests performed on log normalized data (n=4). Error bars represent SE.


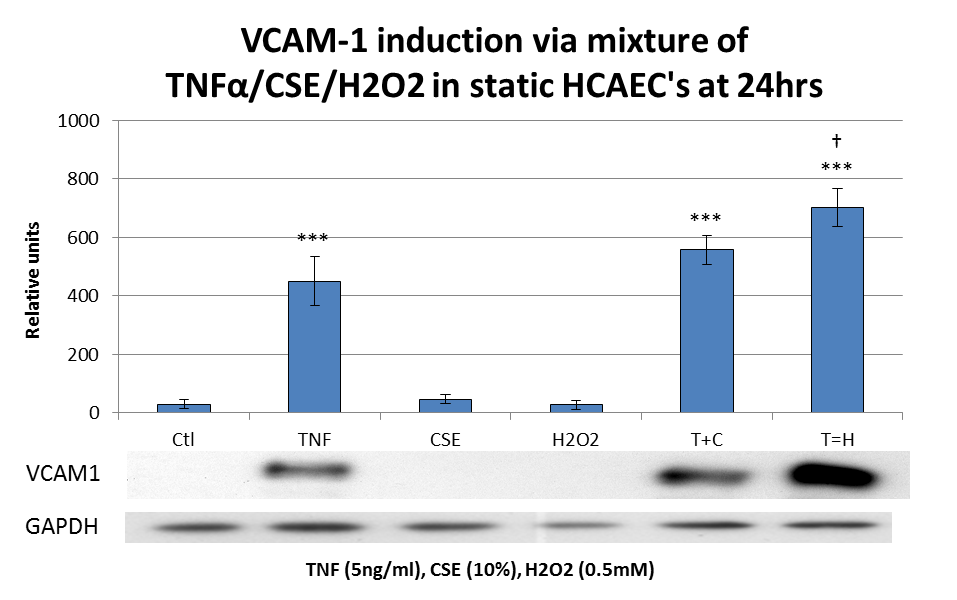


**VCAM1 induction in static HCAECs exposed to a mixture of TNFα/CSE/H_2_O_2_ for 24 hours**

**Relative optical density**

Figure V-2. TNFα (5 ng/ml), CSE (10%), H_2_O_2_ (0.5 mM) and their combination on the induction of VCAM1 protein production in HCAECs at 24 hours. 24hr exposure to TNFα increases VCAM1 in static HCAECs, whereas CSE and H_2_O_2_ did not. Combinations of TNF and CSE (T+C) increased VCAM1 TNF and H_2_O_2_ (T+H) increased VCAM1 production above control and significantly increased VCAM1 above that of TNFα treatment alone. † P<0.05 compared to TNFα; *** P < 0.001 v control. Statistical tests performed on log transformed data (n=4). Error bars represent SE.

#### TNFα, but not CSE, reduces cell number in static HCAECs, but not through a reduction in cell viability.

Before proceeding it was necessary to ensure that cells were not undergoing apoptosis during our experiments. In order to do this several techniques were applied. This included looking at cell number, investigating the expression of markers of apoptosis, and ICC for cytotoxicity.

TNFα is known to both induce and protect cells against apoptosis in tissue culture, depending upon the concentration, time point and manifestation of other protective mechanisms [5-8]. To analyse the effect of TNFα on cell viability, confluent cultures of HCAECs were exposed for 24 hours to different TNFα concentrations (0.5-10 ng/ml). Cell number was significantly reduced with increasing TNF concentration as indicated by Picogreen assay (Figure V-3A), suggesting that there is either an increase in apoptosis or a reduction in proliferation in these cells. However, western blot analysis suggests that PARP cleavage, an indicator of apoptosis, was not significantly increased with TNFα compared to untreated control (Figure V-4A). This indicates that whilst TNFα is known to induce apoptosis, there may be balanced induction of anti-apoptotic mechanisms in static culture. This effect is consistent in concentrations up to 10 ng/ml TNFα.


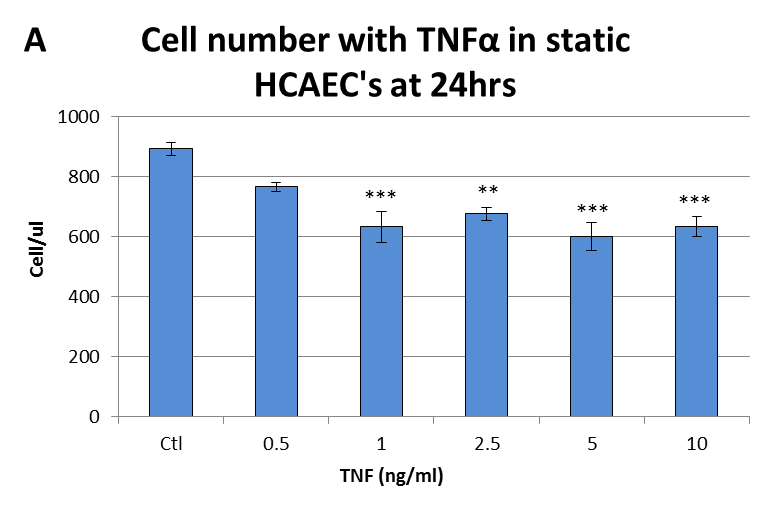

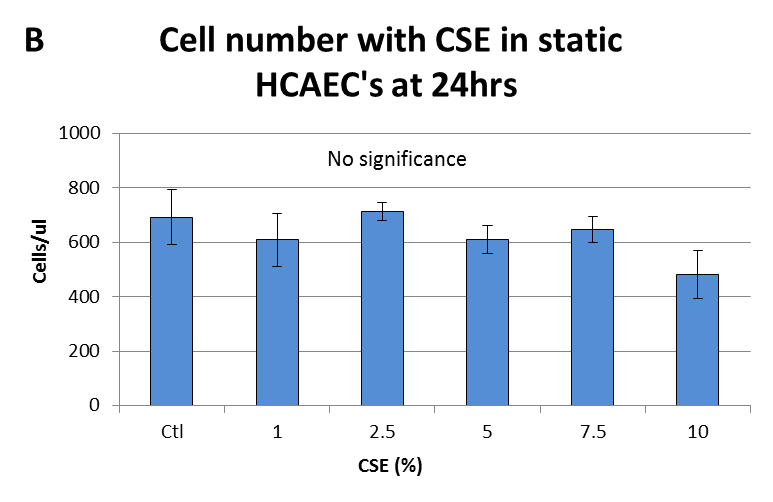


**Cell number in static HCAECs exposed to varying concentrations of TNFα for 24 hours**

**Cell number in static HCAECs exposed to varying concentrations of CSE for 24 hours**

Figure V-3 A: Cell number in endothelial cells exposed to varying concentrations of TNFα for 24 hour, data presented as mean cell number ± SE (n=4). ** P < 0.01, *** P < 0.001 v control. B: Cell number in endothelial cells exposed to varying concentrations of CSE for 24 hours, data presented as mean cell number ± SE (n=4).

CSE is known to induce an immortalised phenotype in some cells in part by influencing apoptosis or perhaps necrotic events [9,10]. Here, exposing HCAECs to CSE for 24 hours had no effect on cell number (Fig V-3B). There was also no significant induction of PARP cleavage in CSE treatment, up to concentrations of 10% CSE (Fig V-4B). However a very low level of PARP cleavage was detected in this experiment amongst all samples, which may be a consequence of culturing endothelial cells in static culture.

Pathological ROS, especially those of CSE, have been demonstrated to induce damage to membranes in several cell types [11,12]. In order to test whether cytotoxicity was induced by treatments, a cytotoxicity assay was used to assess any damage to the cells. Using a live dead cell assay (Figure V-5). HCAECs from 3 different donors were exposed to either 5ng/ml TNFα, 10% CSE or the combination of both. Cells were then assessed visually and comparing treatments to a methanol control known to induce cell death. Neither TNFα, CSE, nor the combination, were found to induce any cytotoxicity above untreated control. Thus all treatments were deemed to have a negligible influence over apoptosis or cellular toxicity.


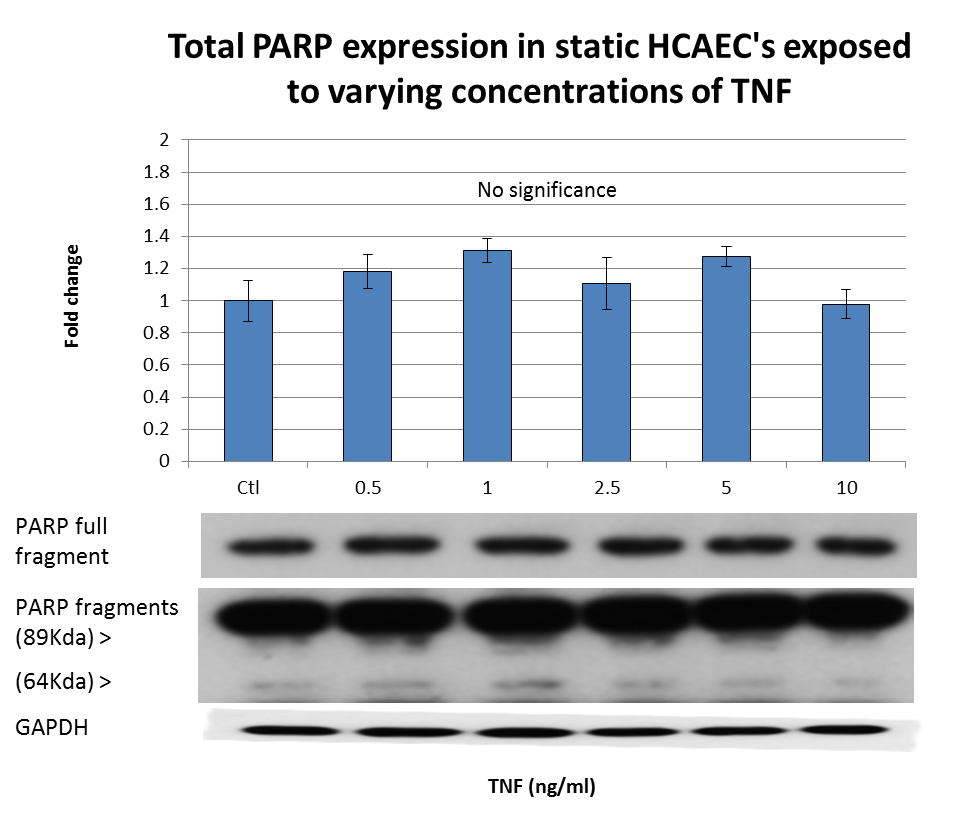

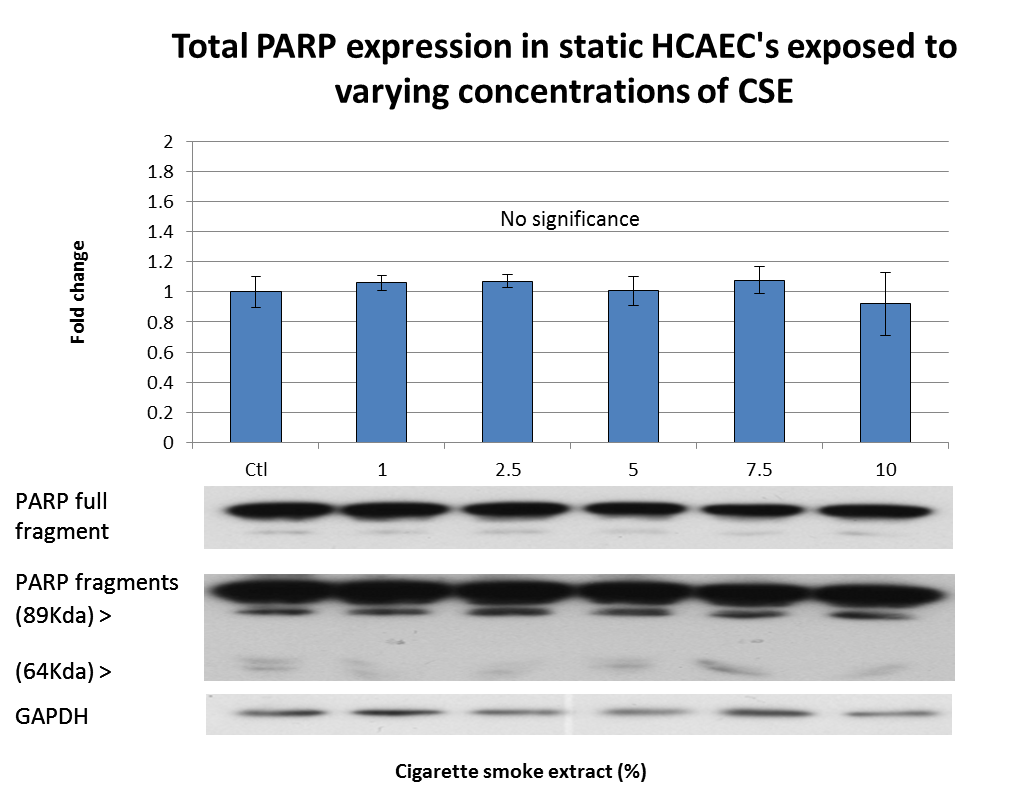


**Total and cleaved PARP expression in HCAECs exposed to varying concentrations of TNFα for 24 hours**

**Relative optical density**

**Relative optical density**

**Total and cleaved PARP expression in HCAECs exposed to varying concentrations of CSE for 24 hours**

**B**

**A**

Figure V-4 A: Total and cleaved PARP expression in HCAECs exposed to varying concentrations of TNFα for 24 hours, data presented as mean fold changes over control ± SE (n=4). Full fragment at 116kDa, cleaved fragments at 89kDa and 64kDa, no significant difference. B: Total and cleaved PARP expression in HCAECs exposed to varying concentrations of CSE for 24 hours, data presented as mean fold changes over control ± SE (n=4). Full fragment at 116kDa, cleaved fragments at 89kDa and 64kDa, no significant difference.

***Live/dead cell assay for cell viability and cytotoxicity***


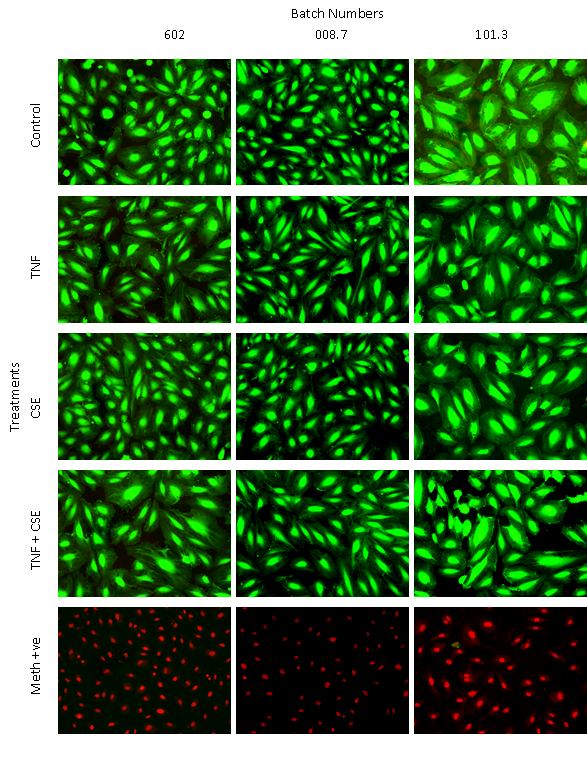


Figure V-5: Live/dead cell assay on 3 separate batches of static HCAECs exposed to either TNFα (5 ng/ml), CSE (10%), or the combination of TNFα and CSE, compared to a methanol control. Green fluorescence of cells indicates healthy and intact cell membranes, whereas red indicates the breakdown of cells resulting from cytotoxicity or death. Toxicity without death can result in both red and green staining.

#### Time Course to assess the temporal induction of NRF2-target gene HMOX1 by CSE.

HMOX1 is a well-established Nrf2 responsive gene. In response to TNFα alone endothelial cells did not produce any detectable HMOX1 (Fig V-6), however CSE treatment resulted in a significant in increase in HMOX1 protein expression, causing a peak 140 fold increase in HMOX1 expression at 16 hours, reducing to 90 fold by 24 hours. This response appeared to be transient, with very low expression of HMOX1 at 8 hours and 48 hours. For this reason it was decided that a 16 hour experimental time point was appropriate for future experiments and that treatment of CSE every 16 hours would maintain maximal Nrf2 activation.


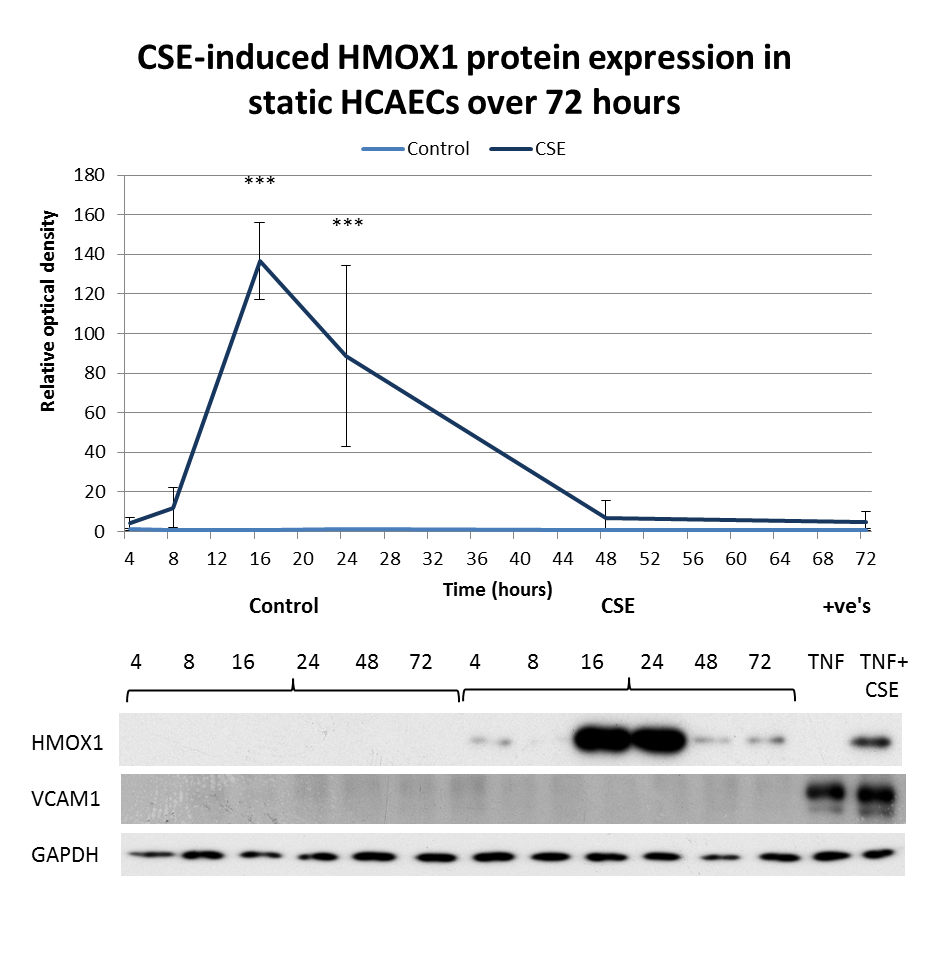


Figure V-6. Time course of HMOX1 production with TNFα, CSE, and combination of TNFα and CSE, in static HCAECs over 72 hours. HMOX1 expression was quantified using western blotting analysis, expressed as mean fold change against control ± SE (n=4), *** P < 0.001 v control.

**References**

1. Hürlimann, D., et al., *Anti–Tumor Necrosis Factor-α Treatment Improves Endothelial Function in Patients With Rheumatoid Arthritis.* Circulation, 2002. **106**(17): p. 2184-2187.

2. Bosello, S., et al., *TNF-alpha blockade induces a reversible but transient effect on endothelial dysfunction in patients with long-standing severe rheumatoid arthritis.* Clinical Rheumatology, 2008. **27**(7): p. 833-839.

3. Donato, A.J., et al., *Role of NFκB in age-related vascular endothelial dysfunction in humans.* Aging, 2009. **1**(8): p. 678–680.

4. Donato, A.J., et al., *Direct Evidence of Endothelial Oxidative Stress With Aging in Humans.* Circulation Research, 2007. **100**(11): p. 1659-1666.

5. Grethe, S., et al., *p38 MAPK mediates TNF-induced apoptosis in endothelial cells via phosphorylation and downregulation of Bcl-xL.* Experimental Cell Research, 2004. **298**(2): p. 632-642.

6. Stehlik, C., et al., *Nuclear Factor (NF)-κB–regulated X-chromosome–linked iap Gene Expression Protects Endothelial Cells from Tumor Necrosis Factor α–induced Apoptosis.* The Journal of Experimental Medicine, 1998. **188**(1): p. 211-216.

7. Dimmeler, S., et al., *Suppression of Apoptosis by Nitric Oxide via Inhibition of Interleukin-1β–converting Enzyme (ICE)-like and Cysteine Protease Protein (CPP)-32–like Proteases.* The Journal of Experimental Medicine, 1997. **185**(4): p. 601-608.

8. Robaye B, et al., *TNF induces apoptosis (programmed cell death) in normal endothelial cells in vitro. .* Am J Pathol, 1991. **138**: p. 447.

9. Narayan, S., et al., *Cigarette smoke condensate-induced transformation of normal human breast epithelial cells in vitro.* Oncogene, 2004. **23**(35): p. 5880-5889.

10. Damiani, L.A., et al., *Carcinogen-Induced Gene Promoter Hypermethylation Is Mediated by DNMT1 and Causal for Transformation of Immortalized Bronchial Epithelial Cells.* Cancer Research, 2008. **68**(21): p. 9005-9014.

11. Niki, E., et al., *Membrane Damage from Lipid Oxidation Induced by Free Radicals and Cigarette Smokea.* Annals of the New York Academy of Sciences, 1993. **686**(1): p. 29-37.

12. Machlin, L.J. and A. Bendich, *Free radical tissue damage: protective role of antioxidant nutrients.* The FASEB Journal, 1987. **1**(6): p. 441-5.
